# Evaluation of the impact of pharmaceutical trainings and tools on the proper use of medicines in pediatrics

**DOI:** 10.3389/fphar.2023.1143974

**Published:** 2023-04-25

**Authors:** F. Charles, A. Castet-Nicolas, C. Amouroux, J. Moreau, O. Werner, D. Morin, P. Berland, M. Fila, G. de Barry

**Affiliations:** ^1^ Department of Clinical Pharmacy, Montpellier University Hospital, Montpellier, France; ^2^ IRCM, INSERM U1194, University of Montpellier, Montpellier, France; ^3^ Paediatric Endocrinology and Mineral Bone Diseases Unit, Montpellier University Hospital, OSCAR Rare Diseases Network, School of Medicine, University of Montpellier, Montpellier, France; ^4^ PhyMedExp, INSERM, University of Montpellier, Montpellier, France; ^5^ Unit of Paediatric Pulmonology and Cardiology, Department of Paediatrics, Montpellier University Hospital, Montpellier, France; ^6^ Paediatric Nephrology Department, SORARE Reference Centre, Montpellier University Hospital, School of Medicine, University of Montpellier, Montpellier, France; ^7^ Department of Public Health, Clermont-Ferrand University Hospital, Clermont-Ferrand, France

**Keywords:** clinical pharmacy, medication error, pediatrics, securing, risk

## Abstract

**Introduction:** After six years of medication errors’ (MEs) collection and analysis in a pediatric unit of a French University Hospital, the number of MEs was no longer decreasing. We then decided to set up pharmaceutical training and tools and evaluate their impact on the occurrence of ME.

**Materials and methods:** This monocentric prospective study was carried out in the form of audits of prescriptions, preparations, and administrations before and after intervention (A1 and A2). After the analysis of A1 results, feedback was given to the teams, some tools for the proper use of medication (PUM) were distributed, and A2 was conducted. Finally, A1 and A2 results were compared.

**Results:** Each audit included 202 observations. A total of 120 MEs were identified during A1 and 54 for A2 (*p* < 0.0001). The observation rate with at least 1 ME decreased from 39.11% to 21.29% (*p* < 0.0001), and no observation had more than two MEs during A2 in contrast to A1 (*n* = 12). Human factors were responsible for the majority of MEs. The audit feedback allowed professionals to feel concerned about ME. The PUM tools received an average satisfaction rating of 9/10. The staff had never participated in this type of training, and all felt it was useful to apply PUM.

**Conclusion:** This study showed a significant impact of pharmaceutical training and tools on the pediatric PUM. Clinical pharmaceutic actions allowed us to reach our objectives and satisfied all the staff. They must, therefore, be continued to limit human factors’ impact and thus contribute to the safety of drug management in pediatrics.

## 1 Introduction

Medication errors (MEs) are responsible for about 5% of hospitalizations. In hospitalized patients, they can lead to an increase in the length of stay, morbidity and mortality, and costs of care (Assiri et al., 2018). The National Coordinating Council for Medication Error Reporting and Prevention (NCC-MERP) defines MEs as any “preventable event that may cause or lead to inappropriate use of the medication or harm to the patient” (National Coordinating Council for Medication Errors, 2014). Pediatrics can be considered an ME risk factor due to children’s physiological specificities, inappropriate dosage forms, lack of pediatric data, or prescriptions outside the marketing authorization. For all these reasons, the rates of MEs in pediatrics are often high: Prot et al*.* and Ozkan et al*.* found 31.3% of MEs in a French university hospital (UH) and 36.5% of MEs in a Turkish UH, respectively (Prot et al., 2005; Ozkan et al., 2011). There are many initiatives to reduce this risk in pediatrics. Maaskant et al*.* compared several interventions to reduce MEs and their consequences on patients: clinical pharmacist (CP) presence in the teams, computerized physician order, barcode medication administration system, use of structured prescribing form, and control checklist (Maaskant et al., 2015). This comparative study does not allow concluding on the effectiveness of these interventions. However, CP presence can help prevent errors in pediatric units. According to Malfará *et al.*, pharmacists can both minimize prescription pharmacological risks in a pediatric intensive care unit and generate savings ($4828 in 1 year) (Malfará et al., 2018). CPs can also prevent 81.3% of MEs according to Fortescue et al*.* acting specifically on prescription errors (Fortescue et al., 2003). Moreover, Wang et al*.* showed that pharmacists can stop 78% of prescription errors but that they have no impact on administration errors which are mainly due to human factors and therefore are most difficult to avoid (Wang et al., 2007).

In Montpellier UH, a pharmacy resident has been working full-time since 2013 in the multidisciplinary pediatric department. A previous study on MEs, presented in congress, was carried out in the department in 2015. This work highlighted 84 MEs over 16 months with 58.3% of reports made by the pharmacist. These MEs were evaluated in a multidisciplinary feedback committee in order to define corrective actions for each ME and avoid their recurrence. This work led to improve awareness on drug iatrogeny and especially MEs among the entire department’s staff. The collection of MEs and semi-annual multidisciplinary feedback committee has been maintained to date. Despite the feedback of this work to stakeholders every six months and pharmaceutical presence, the number of MEs was no longer decreasing. These MEs were mostly related to human factors and therefore seem difficult to prevent with the current methodology. The active participation of a CP in ward helps reduce prescription errors, but this is difficult to act on human factors (Fernández-Llamazares et al., 2012). We wanted to show that this active participation can reduce the occurrence of MEs through training and the creation and dissemination of tools for proper drug use.

## 2 Methods

This prospective, descriptive, and monocentric study was conducted in the multidisciplinary pediatric department of the Montpellier UH (France) divided into two units: pediatric nephrology–endocrinology and pediatric cardiology–pneumology units. Each unit hosts 10 beds. These two units share a team of 21 nurses, 16 caregivers, and a head nurse. The medical staff is composed of 6 pediatric residents and 14 senior pediatricians.

### 2.1 Population studied

Care of children hospitalized in the department during the following periods: 5 November to 9 December 2020 (period audit 1; A1) and 8 March to 19 April 2021 (period audit 2; A2) were analyzed.

The exclusion criteria were the absence of the drug prescription.

### 2.2 Study design

We conducted an initial audit (A1) on medication prescriptions, preparations, and administrations. Results of A1 were analyzed, and the initial rate of ME was determined. After that, audit feedback was provided to the teams in the form of a slide show. It presented main results of the first audit with positive and negative points observed for the different stages of the drug management (DM) audited. It also outlined ways to improve stages with the least amount of compliance. A satisfaction questionnaire on audit feedback was developed and collected. The following data were reviewed: responder position held, seniority, previous participation in an audit feedback, usefulness of the audit feedback to feel concerned, need for tools, and format of tools needed.

The results of this first audit and the satisfaction questionnaire led to develop tools for the proper use of medication (PUM). These documents were validated by an expert hospital pharmacist experimented in pediatrics, the head nurse, three nurses, and two volunteers who were senior physicians. After tool distribution, a satisfaction questionnaire on the tools was drawn up and sent to the team members. The following data were reviewed: awareness of the audit feedback, frequency of reproduction of this type of experience, experience with this type of training, usefulness to the drug PUM, frequency of the use of the tools, and satisfaction with the tools rated out of 10.

A period of tool appropriation (3 weeks) was left before the second audit (A2). A2 was performed to evaluate the impact of the implementation of training sessions and pharmaceutical tools on the occurrence of MEs.

### 2.3 Data collection

Audit data were collected using evaluation tables created specifically for this study.

An observation corresponds to the audit of a drug prescribed to a given patient and a given route of administration. All the steps of the DM (i.e., prescription, preparation, and administration) were audited. With this method of collection, the same prescription for the same patient could be audited several times with a different preparation and administration. All observations were included in the analysis.

Data collection consisted in the following:

• 34 criteria for the parenteral route: 4 for prescription, 15 for preparation, and 15 for administration
• 23 criteria for the oral route: 4 for prescription, 7 for preparation, and 12 for administration
• 18 criteria for the pulmonary route: 4 for prescription, 3 for preparation, and 11 for administration
• 16 criteria for the rectal route: 4 for prescription, 3 for preparation, and 9 for administration.

The audited criteria were defined according to national recommendations of good practice (French High Authority of Health and Ministry of Health), the French Society of Clinical Pharmacy, and the manual of DM of our institution (Qualité, 2012; Outils, 2013; SFAR, 2016). For example, for administration, the common criteria to all routes were adherence to the administration schedule; verification of the patient’s identity; verification of the concordance between the prescription, the drug, and the patient; dose administered; explanations given to the patient; verification of the absence of allergy; traceability of the administration; and hygiene.

Observations took place between 7:00 a.m. and 8:00 p.m. and were realized by five different auditors (pharmacy resident or students). The auditors followed the nurses as they prepared and administered medications on their rounds. Data were collected using a paper audit chart. Prescription data were provided *a posteriori* by the prescribing software (PS) and the computerized patient record.

### 2.4 ME definition

The same methodology was followed for the result analysis of each audit. First, the pharmacy resident rated the compliance of each audited criterion for each observation. Then, he rated presence or absence of MEs based on the auditors’ observations at each step. These MEs were validated by two hospital pharmacists experienced in pediatrics. Thus, an observation could contain several MEs. This is why the rate of ME was defined as the number of MEs (n) divided by the number of observations per step of the DM (N).

Definition and classification of MEs followed those of NCC-MERP (Assiri et al., 2018). Category A concerns circumstances or events that could lead to ME. Category B includes MEs with a drug that did not reach the patient, and category C groups together MEs without consequences for the patient. Category D includes errors occurred that reached the patient and required monitoring. Categories E, F, G, H, and I concern errors with harm of death for the patient. The type of MEs could be as follows:

- Dose omission (e.g., unprescribed, unprepared, or unadministered dose)
- Wrong dosage or concentration (e.g., administration of a 10-mg capsule instead of a 20-mg capsule)
- Wrong nature of drug (e.g., confusion between 5% polyionic and 5% glucose)
- Wrong dosage form (e.g., error between the syrup and granules form)
- Wrong technique of administration (e.g., mixtures of several drugs for administration by a nasogastric tube)
- Wrong route of administration (e.g., per os and not intravenous administration)
- Wrong rate (e.g., administered intravenously directly and not over 20 min as recommended)
- Wrong duration (e.g., prescription of anesthetic pre-medication for 20 days)
- Wrong time of administration (e.g., administered with 4 h delay)
- Therapeutical and clinical monitoring (e.g., traceability of the administration not performed)
- Deteriorated drug error (e.g., pre-prepared, non-stable parenteral drug)
- Other

Types of MEs and potential causes of the errors were assessed by the resident and two hospital pharmacists experienced in pediatrics. The causes’ classification followed NCC-MERP taxonomy: communication, name confusion, labeling, and human factors (Assiri et al., 2018).

The rationale of prescribing was not questioned during audits.

### 2.5 Pharmaceutical trainings

According to A1’s results, two team trainings were conducted by the pharmacy resident: an audit feedback and a presentation of the proposed PUM tools. These training sessions for both doctors and nurses in the department were carried out in different ways depending on the profession. For the nurses, training slideshows were sent by professional messenger for self-training. The satisfaction questionnaires were to be completed online afterward. For physicians, the pharmacy resident presented slideshows to medical staff. The satisfaction questionnaires were distributed during this staff presentation.

### 2.6 Statistical analysis and ethical approval

Descriptive analyses were conducted in full population and in four subgroups of administration routes (parenteral, oral, pulmonary, and rectal).

Data were presented as numbers and percentages or means+/-standard deviation (SD).

Qualitative variables were compared using the chi-2 test (or Fisher’s exact test if theoretical numbers were less than 5). Quantitative variables were compared using the Mann–Whitney test. For all the tests performed, the risk of consented error was set at 5%. Statistical analyses were performed with SAS v9.4 software^®^.

This study was approved by the Institutional Review Board of Montpellier University Hospital (IRB number: 202000620).

## 3 Results

### 3.1 Audit characteristics

Department activity and patients’ characteristics for both audits are described in Table 1. During A2, the unit activity was more important than during A1 with more admissions and higher bed occupancy. The patient mean age was similar for both audits, but patients during A2 were less medicated.

**TABLE 1:** Audit descriptive characteristics.

	Audit 1 (A1)	Audit 2 (A2)	*p*
Number of admissions, *n*	119	209	
Number of admissions per day, *mean* (*SD* *^a^*)	3.51 (1.84)	4.86 (1.64)	*0.0024*
Bed occupancy rate, *mean* (*SD* *^a^*)	0.80 (0.16)	0.83 (0.18)	*0.0075*
Patients age-years, *mean* (*SD* *^a^*)	7.18 (6.65)	7.30 (5.71)	*0.6708*
Number of prescriptions lines per patient, *mean* (*SD* *^a^*)	9.58 (5.04)	7.53 (4.98)	*<0.0001*
Mean duration of stay in days, *mean* (*SD* *^a^*)	12.83 (7.24)	9.55 (9.86)	*<0,0001*
Length of service of the audited staff in years, *mean* (*SD* *^a^*)	5,79 (4,32)	3,93 (3,89)	*<0,0001*
Number of observations, *n*	202	202	
Number of different prescribed drugs, *n*	91	93	

^a^ SD, standard deviation.

Each audit was conducted with 20 days of actual observation and consisted of 202 observations.

The pharmacy resident was the lead auditor and performed 94.6% of the observations for A1 and 89.11% for A2 (*p* = 0.0075).

### 3.2 Medication errors’ characterization

#### 3.2.1 General data

Herein, 120 and 54 MEs were recorded during A1 and A2, respectively .

#### 3.2.2 Data by stage of the drug management in the care unit

The evolution, according to the steps of the DM, of the number of MEs and the ME rate are summarized in Table 2. The prescription step involved less ME than other steps (15% of ME for A1 and 9.25% for A2). The preparation step was the most concerned by ME for A1 with 65% of ME and showed a significant decrease between the two audits. Concerning the administration step, this step involved 20% of ME for A1 and 44% for A2, but this rise was not significant. In all, there was a significant decrease in the ME rate between the two audits.

**TABLE 2:** Evolution of the number and rate of medication errors between the two audits by stages of the drug management of MEs between two audits by stages of DM.

	Medication error Audit 1 (A1)	Medication error Audit 2 (A2)	
Stage	*n* (%^a^)	*n* (%^a^)	*p*
Prescription (*N* = 202)	18 (8.91)	5 (2.48)	*0,0052*
Preparation (*N* = 202)	78 (38.61)	24 (11.88)	*<0,0001*
Administration (*N* = 202)	24 (11.88)	25 (12.38)	*0,8788*
Total (*N* = 606)	120 (19.80)	54 (8.91)	*<0,0001*

^a^ Percentage = *n*/number of observations per step (*N*).

#### 3.2.3 Data per observation

We showed that several MEs could concern the same observation (up to 4). The number of MEs per observation is presented in Table 3. Of the A1 observations, 39.11% had at least one ME, and this rate decreased significantly during A2 to reach 21.29% (*p* = 0.0001). During A2, no observation has more than two MEs unlike A1. The distribution of MEs by observation was significantly different between the two audits. The average of ME decreased significantly between A1 and A2.

**TABLE 3:** Number of observations according to the number of ME and number of ME by observation for audits 1 and 2.

Observation	Audit 1 (A1)	Audit 2 (A2)	*p*
	(*N* = 202)	(*N* = 202)	
	*n* (%)	*n* (%)	
Without ME	123 (60.89)	159 (78.71)	*<0,0001*
With 1 ME	53 (26.24)	32 (15.84)	
With 2 ME	14 (6.93)	11 (5.45)	
With 3 ME	9 (4.45)	0 (0.00)	
With 4 ME	3 (1.49)	0 (0.00)	
Number of MEs by observation, *mean* (*SD* *^a^*)	0.59 (0.91)	0.27 (0.55)	*<0.0001*

^a^ SD, standard deviation.

#### 3.2.4 Nature of MEs

**FIGURE 1:**
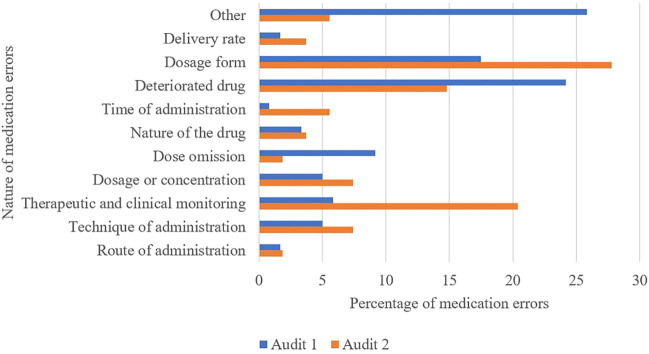
Distribution of medication errors by nature during both audits.

Figure 1 shows the type of the MEs by audit. For audit 1, it was mainly type “other,” and for the second, it was dosage form errors.

The type “other” includes 25 homogenization errors (no homogenization after reconstitution or dilution), 5 hygiene errors (jewelry or lack of hand washing), and 1 non-compliance with the prescription error for audit 1. For audit 2, it was one hygiene error, one administration by unqualified staff (unsupervised student nurse), and one use of a drug without data in children in the literature.

#### 3.2.5 Comparison of causes of error between A1 and A2

Majority of MEs were caused by human factors (95.00% vs. 85.19%) (*p* = 0.0277). Other reasons were the following:

• Packaging or design problem (e.g., non-unitary packaging): 2.5% vs. 11.11%
• Labeling or information problem (e.g., prescription not present on the care plan): 1.67% vs. 0%
• Name confusion (e.g., prescribing magnesium carbonate instead of magnesium chloride): 0.83% vs. 1.85%
• Communication problem (e.g., medication already administered by the caregiver): 0% vs. 1.85%

**FIGURE 2:**
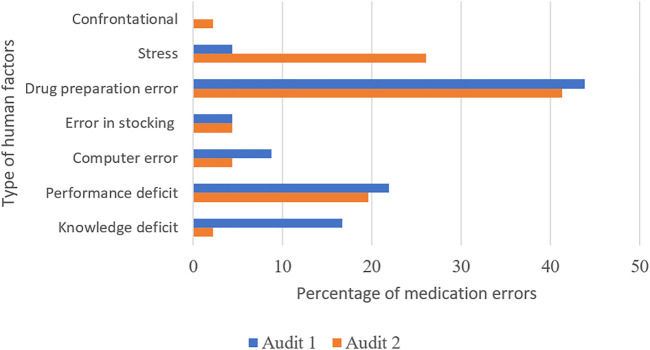
Details of medication errors caused by human factors.

The details of the human factors are presented in Figure 2. Drug preparation errors were the most common errors before performance deficit.

#### 3.2.6 Severity of MEs

For category A, five ME (4.17%) were recorded for A1 and none for A2. MEs with a drug that did not reach the patient (category B) were at the number of 5 (4.17%) for A1 and 3 (5.56%) for A2. Finally, MEs without consequences for the patient (category C) were 110 (91.67%) for A1 and 51 (94.44%) for A2 MEs.

Thanks to the pharmacist’s intervention, there were no ME for categories D, E, F, G, H, and I. Indeed, auditors interrupted the procedure if an observation could lead to consequences for the patient. These interruptions were as frequent during A1 as A2 (three in each audit). For A1, we intercepted an overdose and two advance preparations for unstable drugs. During A2, we stopped two galenic errors for drugs with a narrow therapeutic range and one error of drug’s nature.

### 3.3 Training course: Audit feedback and provision of tools

All team members have been trained.

#### 3.3.1 Audit feedback

The feedback after A1 emphasized the need to consider the following points:

• Parenteral route: Check reconstitution and dilution solvent of each drug.
• Oral route: Administrate anesthetic premedication only if it is prescribed in the PS. Check for the possibility of crushing a tablet or opening a capsule. Make sure to keep an identification of the medication until the patient administration.
• Pulmonary and rectal routes: Administrate anesthetic premedication only if it is prescribed in the PS. Respect the prescribed carrier gas for an aerosol.

We then suggested different areas of improvement about each step of the DM, which are exposed in Table 4.

**TABLE 4:** Areas of improvement proposed in the audit feedback.

Prescribing step	Prescribe doses that can be prepared or administered
	Write a prescription for injectable drug specifying the dilution solvent, the volume of drug to be withdrawn, and the administration rate
	Prescribe premedication for anesthesia
	Refer to the institutional guide for the use of dry forms in order to
	- Check that a tablet is scored or crushable
	- Know if a capsule can be opened and if the enteral route is possible to use a dosage form adapted to the patient’s abilities
Preparation step	Systematically check the condition and expiration date of medication
	Check the methods of reconstitution, dilution, infusion, grinding or opening, potential incompatibilities, and the stability of prepared drugs
	Perform gentle post-reconstitution and post-dilution shaking for injectable preparations
	Separate parenteral and oral treatments prepared to avoid confusion (similar syringes used)
	Identify prepared doses
Administration step	Inform the patient and/or his parents of treatment administration
	Systematically conduct administration tracing as soon as possible post-administration

Of the 40 audit feedback questionnaires handed out, 30 were returned. Among the respondents, 70% had never participated in an audit feedback. This rate was 85% among nurses. The presentation of the audit feedback allowed 94% of physicians and 69% of nurses to feel “completely” concerned.

The audit feedback questionnaire highlighted that some tools for the PUM were required. A table on injectable forms was the most requested tool by all trades. Only the physicians needed protocols in PS. Physicians primarily needed computer-based tools whereas nurses preferred paper-based tools.

Following audit feedback and staff’s responses to the satisfaction questionnaire, the PUM tools were developed.

#### 3.3.2 Tools for the proper use of medicines

According to the results of the first audit and the audit feedback, nine PUM tools were developed or have been made available to the department’s staff (Table 5). The staff was interviewed about these tools through a questionnaire. Of the 38 professionals in the department, 19 had responded to the questionnaire on PUM tools, i.e., 50% response.

**TABLE 5:** PUM tools developed.

Tool	Description
Guide of injectable forms	Table with the injectable drugs most used in the department, reconstitution methods, dilution methods, stability, mode of administration, and major incompatibilities	
Y-compatibility table	Double entry table made available to the services without modification of the initial document from the Geneva UH (Recommandations d’utilisation, 2021)	
Oral forms guide	Table with the most commonly used medications, specifies main simplified indications, method of storage, specific delivery methods outside of the hospital, advice on how to take them, crushable or openable notion, possibility of using an enteral tube, and possible alternatives	
Institutional guide for the dry forms’ uses	Institutional booklet made available in the department, in treatment rooms, and medical offices	
Liquid oral form guide	Table for the oral solutions frequently used in the department with the duration and method of conservation, mL-mg, or drop-mg equivalences and administration device if it was available in the box	
Liquid oral form label	Label used to notify when the bottle is opened and when it should no longer be used	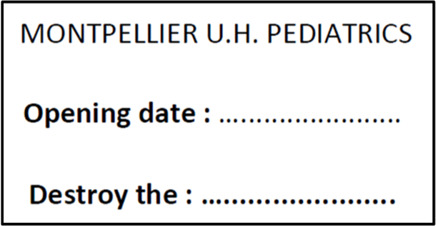
Injectable preparation label	Label ready to use so that nurses can fill in essential information for correct identification of an injectable preparation	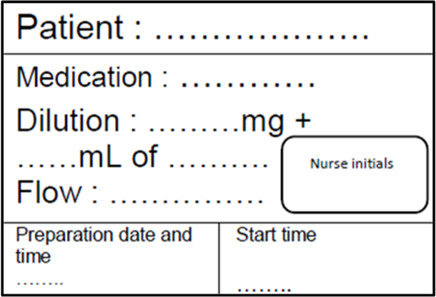
Rinse label	Label available in two types depending on preparation: NaCl 0.9% or glucose 5%	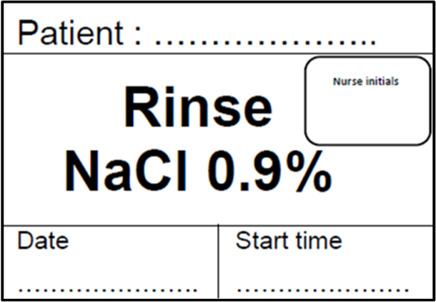
Administration protocol of enoxaparin	Explains the mode of dilution of enoxaparin for the low doses	

All respondents were aware of the audit feedback. All the participants had never had this type of training, and they all found it useful for the PUM. In addition, everyone thought that the experience was one to be repeated. When asked, “How often should an experience of this type be replicated?,” 75% of nurses responded annually, 50% of physicians responded annually, and the other half responded every 2–5 years.

Each tool was to be rated out of 10. The mean satisfaction score of the tools was 9/10.

**FIGURE 3:**
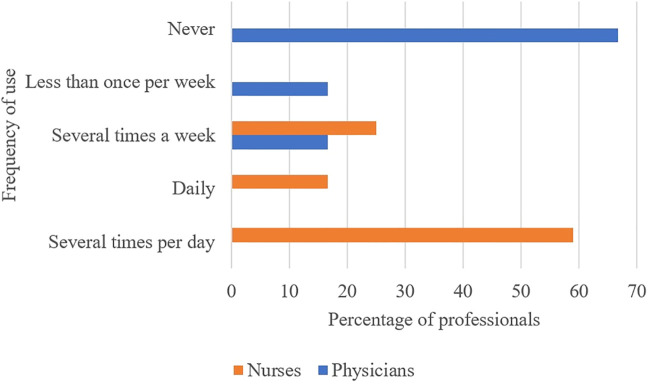
Frequency of use of the proper use of medicines tools by the team by position.

Tools were used more frequently by nurses than physicians, as shown in Figure 3. Majority of doctors had never used tools whereas the nurses used the tools at least several times a week.

## 4 Discussion

We conducted two audits of the three stages of DM in a multidisciplinary pediatric department in a French UH. To the best of our knowledge, this is the first “before-and-after” study performed exclusively in a conventional pediatric unit (excluding intensive care and neonatal units).

The methodology of the two audits can be compared because they have the same number of observations over the same number of days, in the same department and with the same medical and paramedical teams. During the A2 period, service’s activity was more sustained in connection with a bronchiolitis epidemic, which resulted in a significant increase in the average bed occupancy and a higher number of daily admissions. Staff workload had therefore increased between the two audits. Along with this increased activity, patients were significantly less medicated during A2. This is certainly due to the essential non-drug management of patients with bronchiolitis. The mean age of patients was not significantly different between the two audits (7.18 vs. 7.3). It points out that our study included a typical population of the service since there were no neonates. Furthermore, this mean age is a relatively rare finding in the pediatric ME literature. Indeed, studies on ME in children often take place in neonates (Morriss et al., 2009; Stavroudis et al., 2010; Alomari et al., 2015). The seniority of the audited staff decreased significantly between the two audits. According to the Alomari review, there is a ME risk factor like workload (Outils, 2013). Without intervention, we would therefore expect to see an increase in MEs.

Despite all, a significant decrease in the overall ME rate between the two audits was brought out.

The decrease in prescribing MEs may be due to the reduction in patient medication, thus resulting in physicians prescribing workload. We can also hope that this improvement in prescribing is due to the awareness of prescribers through the audit feedback. The PUM tools have probably not had an impact on this improvement as they are mainly intended for nursing practice and have been used little by doctors. During A1, 10 prescribing MEs induced 22 preparation MEs, whereas during A2, 2 prescribing MEs generated a single preparation ME. Decrease in prescribing MEs (18 vs5) therefore directly induced a decrease in preparation ME. Thus, it is essential to have exhaustive feasible prescriptions containing information for preparation to secure downstream steps. A work of prescriptions’ protocolization in the PS is thus to be conducted to facilitate the work of prescribers and thus secure the work of nurses. However, it can also generate new MEs to take into account (like wrong dose and wrong route), and must be adapted to pediatric, as shown by the works of Upperman et al. (2005) and York et al. (2019). As a result of this work, new settings or modifications in the PS have been made to facilitate the prescribers’ work and secure the prescription. However, this configuration is time consuming and depends on the editor. In addition, since our PS is common to both adult and pediatric services, our parameterization capacity is limited.

Concerning the drug preparation step, we observed a significant decrease of ME. Use of conform reconstitution or dilution solvent, post-reconstitution or post-dilution shaking, and also application of the developed labels were more frequent during A2 and could explain this decrease. Preparation is strongly impacted by our training and tools on the PUM. Our tools are widely used by nurses and therefore seem to be adapted to their daily practice of preparing medications The portion of it's MEs during administration step A2. This increase may be due to the increased occupancy of the unit and high patient turnover. Because of this increased workload, we have seen administration of doses that do not comply with those prescribed, a failure to respect the administration schedule, co-administration of oral medications that should not be co-administered, or a lack of administration’s traceability. Decrease in A2 patients’ medication did not offset negative effects of this over activity. Training of nurses on administration schedules for certain molecules and physicochemical interactions could raise awareness on importance of these parameters and thus avoid MEs even in case of work overload. Finally, a reminder must be made on regulatory obligation of administration’s traceability and its importance in ME’s prevention. Our tools have not allowed us to raise awareness on these aspects and must therefore be improved or completed by new trainings.

As Maaskant *et al.* pointed out, the heterogeneity of definition of ME and ME rates make the studies’ comparison difficult (Maaskant et al., 2015). For Prot *et al.,* the ME rate corresponds to the number of ME divided by the sum of the observed and omitted administrations. For Ozkan *et al.* this rate is defined by the number of doses with ME divided by the sum of the observed and omitted administrations. Our rates of observations with ME (39.11% for A1 and 21.29% for A2; *p* < 0.0001) are closed to the published rates of 31.3% and 36.5% by Prot et al. (2005) and Ozkan et al.( 2011), respectively, while our definition was different. Indeed, we calculated the rate of observations with at least one ME. It therefore seems essential to standardize the definitions of ME rates.

By classifying observations by number of MEs, we notice that our work significantly decreased the number of MEs per observation and removed observations with three or more MEs. Decrease in MEs’ number per observation can be explained by the decrease in MEs of prescription (18 vs. 5), which induces a decrease in downstream step MEs.

The ME nature was different between A1 and A2. The decrease of nature “other” in A2 can be enforced by the reminder during the audit feedback of the importance of the preparations’ homogenization. The increase in dosage form errors and monitoring errors during A2 can be explained by lack of attention due to the increased activity of the department. There was no prevalent and/or recurring ME nature from one audit to the next. In studies by Ozkan *et al.* and Prot *et al.*, the most common type of ME was the timing error. In our work, these MEs are very minor with 0.83% of ME in A1 and 5.56% in A2. This difference can be explained by our ME rating methodology. We scored MEs only as administration timing non-compliances that could be clinically significant (such as vancomycin, immunosuppressant, or premedication lag).

Human factors were responsible for most MEs (95% for A1 and 85.19% for A2). During A2, nurses’ workload was increased as compared to A1, which is recognized as a risk factor for ME increasing (Alomari et al., 2015). However, ME proportion related to human factors was lower for A2. This may illustrate a positive aspect of audit feedback on these MEs.

It is important to note that MEs identified during our study never had a significant impact on patient and would certainly have gone unnoticed without this work and therefore unreported. This low severity of our MEs is explained by auditor’s intervention in six MEs to preserve patient safety. These interventions were possible because of the pharmacological knowledge of the auditors (pharmacy resident or student). We did not find such interceptions in the literature. The low patient impact of the MEs found in our study should not be overlooked. Indeed, we know that it is the accumulation of inconsequential MEs and near misses that can lead to serious adverse events. Thus, if the tools and trainings have made it possible to reduce MEs and the number of MEs per act, they have certainly made it possible to reduce the risk of serious adverse events. The PUM tools can allow securing the patient’s care.

The collection method based on observation may be a bias. Observation of staff may influence auditee behavior (Hawthorne and Halo effects) and/or change attitudes due to fear of judgments or in relation to self-questioning (Boet et al., 2012). With 202 observations for each audit on 20 different days, we can expect this bias to be faded. Moreover, another bias may be the five different auditors. To reduce this bias, pharmacy students were trained by the pharmacy resident who was the project leader. The lead auditor was this intern and verified the students’ data collection. In addition, students’ observations have a small impact on our results (5.4% of A1 observations and 10.89% of A2 observations). Another bias of this study is that the department’s pharmacy intern, creator of the audit grid and training materials, was the primary auditor. Unfortunately, this bias cannot be measured but remains the same between the two audits. In our methodology, the same prescription could be audited several times. It could be a bias in the analysis of prescription errors. We can consider that it is not a bias in the analysis of preparation and administration errors since these steps are carried out in a different way and by different nurses in our observations. This type of “before and after” study is difficult to replicate. Indeed, in addition to being observation-based, the assessment of MEs and their classification is subjective. We chose to carry out this assessment in a collegial manner with a pharmacy intern and two pharmacists experienced in pediatrics to reduce this subjectivity. A pharmacy or medical intern stays for six months in the department. To limit auditor bias and staff changes, we chose to conduct the entire study in less than 6 months. This tight schedule did not allow for a long phase of appropriation of the trainings and tools by the teams. This short interval does not allow prejudging the longevity of the implemented actions.

The audit feedback allowed us to remind a set of good practice rules and to make teams aware of ME identified during A1. Staff satisfaction regarding this feedback was assessed through a questionnaire. It was observed that 70% of respondents had never taken part in the feedback audit. This *a priori* risk management method does not seem to be widely used in our institution.

Following the audit feedback, professionals were willing to participate in a presentation of the PUM tools. Awareness was therefore important. This underlines the favorable climate for our work in this unit. It is important to remember that since 2015, in this department, semi-annual multidisciplinary feedback committee on MEs takes place. This ongoing awareness of the importance of EM control should be taken into account when comparing the results of this study to those of others.

Regarding the format of desired PUM tools, a difference between physicians and nurses is observed. Physicians mainly wanted computer-based tools, and nurses needed handwritten tools. We wanted to respect this double desire by making all the tools available in both electronic and paper form.

The staff satisfaction to PUM tools was assessed through a questionnaire. Staff had never had this type of training and found it useful for the PUM. Such an experiment should therefore be repeated at most every year, or even every 2 to 5 years. This period is a good compromise to maintain the vigilance of the teams, to make newcomers aware of the situation, and to update the tools. It also avoids having an action repeated very frequently which would decrease team’s motivation.

## 5 Conclusion

The methodological scheme followed in this study (before/ after intervention) remains rare in the literature regarding the PUM in pediatrics. We confirmed the importance of human factors in the occurrence of ME and demonstrated a significant impact of pharmaceutical training and tools on safety of DM. The most significant result was found for the preparation stage, underlining major influence of training and tools on the paramedical staff. Training courses have satisfied all staff who continues to request clinical pharmacy activities. This work shows the importance of the presence of a CP in the unit for the safety of DM, thanks to the daily awareness of the inherent risks to the teams. This presence would make it possible to maintain the actions implemented and their effects by ensuring the updating of tools and the training of new team members. Therefore, the pharmaceutical presence in the unit must be continued.

## Data Availability

The raw data supporting the conclusion of this article will be made available by the authors, without undue reservation.

## Abbreviations

A1, audit 1; A2, audit 2; CP, clinical pharmacist; DM, drug management; ME, medication error; NCC-MERP, National Coordinating Council for Medication Error Reporting and Prevention; PS, prescribing software; PUM, proper use of medication; UH, university hospital.
